# Intelligence

**Published:** 1807-04

**Authors:** 


### INTELLIGENCE.

On Monday, the 9th of March, 1807, the Medical Society of London held their Anniversary Meeting, which was very numerously attended.

Dr. ADAMS having been appointed to deliver the Anniversary Oration, commenced exactly at half past three. The subject he chose excited more than ordinary attention, and his manner of treating it was not less new. Epilepsy has been so long consigned to empiricism, that a learned body was little prepared to hear it taken up by an experienced practitioner. The Orator began by lamenting that the great uncertainty of success had induced the faculty, too generally, to consider Epilepsy as an incurable disease, without attending to those varieties in its cause which should influence the treatment. He regretted that the distinction of *chronic* and *acute* Epilepsy, preserved from Hippocrates to Cælius Aurelianus, should have been so little noticed by the moderns; and proposed a sub-division of each of these forms of the disease into *periodical* and *habitual*. He remarked that these divisions are not to be considered like some of the classifications of Nosologists, which cannot always be ascertained; and, if ascertained, would no way influence our practice; but that the want of such distinctions in Epilepsy, had made us reject some useful remedies, because they were not uniformly serviceable, and over value others from a success which was at least doubtful. That a treatment, absolutely necessary in acute Epilepsy, would be highly improper in the chronic, and that the remedy for periodical Epilepsy could never be applied to the habitual. We shall not enter further on the subject, because the treatment is so entirely connected with the Author's opinions of the various causes of the disease, that it would be injustice to mention one, whilst the limits of our work must must prevent the other, and also because we have reason to believe that the Oration will be printed.

The Register then read the List of Officers and Council for the year ensuing, as follows:

OFFICERS AND COUNCIL OF THE
MEDICAL SOCIETY OF LONDON.

Sims, James, M. D. PRESIDENT.
Walker, Sayer, M. D. TREASURER.
Hurlock, Joseph, S. LIBRARIAN.
Good, John Mason, S. SECRETARY.
Hamilton, James, M. D. SECRETARY.
Poignand, L. M. D. SEC. for foreign Correspondence.

Hulme, Nathaniel, M. D.
Ford, Edward, S.
Bancroft, Edward, M. D.
Andree, John, M. D.
Jackson, Joseph, A.
Chamberlaine, William, S.
Clough, Henry Gore, M. D.
Ridout, John Gibbs, S.
Hooper, James Hill, S.
Ware, James, S.
Hayes, Sir John Macnamara, Bart. M. D.
Webb, Thomas, S.
Newby, Charles, M. D.
Ring, John, S.
Bradley, Thomas, M. D.
Seaton, James, A.
Kilpatrick, George, A.
Thornton, Robert John, M. D.
Griffiths, Samuel, A.
Heaums, Peter, A.
Robinson, Henry, A.
Pearson, Richard, M. D.
Clutterbuck, Henry, M. D.
Headington, William, S.
Taunton, John, S.
Lewis, William, S,
Macdonald, William Rio, A.
Atkinson, Benjamin, A.
Pinckard, George, M. D.
Hopkins, W. L. A.
Cordell, W. Daniel, S.
Leese, Lewis, S.
Harvey, Ludford, S.
Adams, Joseph, M. D.
Temple, Richard, M. D.

ANNIVERSARY ORATION for next Year, Birkbeck, Geo. M. D.
Wagstaffe, Mat. French, A. REGISTER.

The Prize Questions for the Fothergillian Medals were then read, for which see our Journal, No. xcvi, p. 198.

---

It is with much satisfaction we inform our readers, that the island of Madeira is now likely to afford all that relief to consumptive, scrofulous, and asthmatic patients, which the bounty of heaven seems exclusively to have destined for that spot. The difficulty so long complained of, and so universally lamented, the want of proper accommodation for strangers, is now removed. Three houses are open, two by English, and one by a Portuguese long accustomed to English manners, for receiving invalids; and houses are erected for families in sufficient number to produce an useful competition. It is unnecessary to add any thing on the salubrity of Funchall for a winter's residence, or on the beauties of the higher parts of the island, and their refreshing coolness during the summer months.

M. LE

M. LE GRANGE has recently examined the substance called tannin, the character of which is to form with gelatine an insoluble compound; he finds in it an affinity for alkalies, the earths, and metallic oxydes, and the faculty of converting itself into gallic acid by absorbing oxygen.

Dr. CHARLES FOTHERGILL is now engaged in preparing a work for the press, which can scarcely fail to excite very general interest. With a view of clearing up some doubtful points in the Zoology of Great Britain, he last spring made a voyage to all the northern isles, comprehending the Orcades, Shetland, Fair Isle, and Fula, and remained amongst them during the greatest part of the year, employed in the investigation of their natural history, antiquities, state of their agriculture and fisheries, political importance, manners, customs, condition, past and present state, &c. &c. A general and particular account of which will shortly be given to the public, accompanied by maps and numerous engravings; containing the fullest and completest description that has been published of those remote and hitherto neglected regions.

Mr. COOPER's First Lines of the Practice of Surgery, complete in one volume octavo, will make its appearance early in April, accompanied by essential plates, at a moderate price, not exceeding twelve shillings.

The Summer Course of Dr. BADHAM's Lectures on the Practice of Physic, Chemistry, and Materia Medica, will be commenced on Monday, the 4th of May.

Dr. REID will commence his Summer Course of Lectures on the Theory and Practice of Medicine, on Wednesday, the 6th of May.

